# Borohydride Synthesis of Silver Nanoparticles for SERS Platforms: Indirect Glucose Detection and Analysis Using Gradient Boosting

**DOI:** 10.3390/s25134143

**Published:** 2025-07-03

**Authors:** Viktoriia Bakal, Olga Gusliakova, Anastasia Kartashova, Mariia Saveleva, Polina Demina, Ilya Kozhevnikov, Evgenii Ryabov, Daniil Bratashov, Ekaterina Prikhozhdenko

**Affiliations:** Science Medical Centre, Saratov State University, 83 Astrakhanskaya Str., 410012 Saratov, Russia; bakalva@sgu.ru (V.B.); guslyakovaoi@sgu.ru (O.G.); kartashovaam@sgu.ru (A.K.); savelievams@sgu.ru (M.S.); ryabovea@sgu.ru (E.R.); prikhozhdenkoes@sgu.ru (E.P.)

**Keywords:** non-woven materials, polyacrylonitrile, in situ AgNP synthesis, SERS sensors, glucose detection

## Abstract

In recent years, non-invasive methods for the analysis of biological fluids have attracted growing interest. In this study, we propose a straightforward approach to fabricating silver nanoparticle (AgNP)-coated non-woven polyacrylonitrile substrates for surface-enhanced Raman scattering (SERS). AgNPs were synthesized directly on the substrate using borohydride reduction, ensuring uniform distribution. The optimized SERS substrates exhibited a high enhancement factor (EF) of up to 10^5^ for the detection of 4-mercaptobenzoic acid (4-MBA). To enable glucose sensing, the substrates were further functionalized with glucose oxidase (GOx), allowing detection in the 1–10 mM range. Machine learning classification and regression models based on gradient boosting were employed to analyze SERS spectra, enhancing the accuracy of quantitative predictions (R^2^ = 0.971, accuracy = 0.938, limit of detection = 0.66 mM). These results highlight the potential of AgNP-modified substrates for reliable and reusable biochemical sensing applications.

## 1. Introduction

Diabetes mellitus continues to be a major global health challenge in the 21st century, with its prevalence and associated complications placing a significant burden on healthcare systems worldwide. According to the International Diabetes Federation, over 537 million adults aged 20–79 are affected by this disease, and, by 2045, this number is projected to increase to 783 million [1]. Moreover, approximately 240 million people with diabetes are currently unaware of their condition and the complications associated with it, such as cardiovascular disease [2,3], nephropathy [4,5,6], neuropathy [7], and retinopathy [8], all of which contribute to reduced quality of life and elevated mortality rates.

Based on these statistics, there is a pressing need for accessible, reliable, and affordable glucose monitoring technologies. In particular, technologies that are non-invasive and easy to use would be highly beneficial [9,10]. Traditionally, methods of determining glucose levels have included electrochemical glucometers [11], which are based on measuring the current generated during the oxidation of glucose by the glucose oxidase (GOx) enzyme [12]. While these devices offer portability and rapid results, they require frequent blood sampling and are susceptible to interference from hematocrit levels, oxygen tension, and other blood components [13].

To address these limitations, research has advanced toward the development of non-enzymatic electrochemical sensors, such as those utilizing screen-printed electrodes modified with transition metal hydroxides (for instance, Ni(OH)_2_, Cu(OH)_2_, and their composites), which have demonstrated high sensitivity and low detection limits in complex biological samples [9,14]. Spectrophotometric methods, including the hexokinase assay [15,16], are highly accurate but require laboratory infrastructure and time-consuming sample preparation.

In recent years, the focus has shifted toward innovative optical and fluorescence-based sensors, which exploit the unique properties of organic dyes, carbon dots, and functional nanomaterials to achieve sensitive and selective glucose detection [17,18,19,20]. Non-invasive optical techniques, such as Raman spectroscopy, photonic crystal sensors, and impedance-based devices, are actively being developed, although challenges related to sensitivity, selectivity, and reproducibility in real-world applications remain [9,21,22].

Among these, surface-enhanced Raman scattering (SERS) has emerged as a particularly promising technology for glucose sensing [23,24,25]. SERS offers the ability to obtain molecular structural information with high sensitivity, even in aqueous environments, due to its low sensitivity to water interference. However, the inherently weak Raman signal of glucose poses a challenge for direct detection [26]. To overcome this, indirect detection strategies are often used, such as the glucose reaction with an enzyme (glucose oxidase, GOx), leading to the reduction of GOx and an increase in the concentration of byproducts (for instance, hydrogen peroxide), which can be sensitively monitored using SERS-active nanostructures [27,28].

The design of modern SERS sensors often leverages advanced polymeric materials as substrates, including both natural and synthetic polymers, to optimize sensor performance and stability. Among natural polymers, chitosan stands out due to its excellent biocompatibility, biodegradability, and film-forming properties. Chitosan’s abundance of amino and hydroxyl groups enables strong interactions with metal nanoparticles and biomolecules, facilitating the effective immobilization of enzymes such as glucose oxidase and enhancing the sensitivity and selectivity of SERS-based biosensors [29,30]. Additionally, chitosan’s inherent antimicrobial properties can help to maintain sensor integrity in biological environments.

Polystyrene is another popular choice due to its hydrophobicity, mechanical robustness, and ease of fabrication into various forms, such as beads, films, or fibers. Its inert nature minimizes non-specific interactions and background signals, which is particularly important for SERS applications where signal clarity is paramount. Polystyrene substrates can be easily functionalized or coated with metal nanoparticles, providing a stable and reproducible platform for SERS enhancement [31].

Polyacrylonitrile (PAN), known for its high chemical and thermal stability, is frequently utilized as a support material in the form of non-woven mats or electrospun fibers. PAN’s surface can be readily modified to anchor metal nanoparticles, creating a high-surface-area substrate that is ideal for SERS applications [32,33,34]. Its resistance to degradation in harsh chemical environments ensures the long-term durability of the sensor, while its flexibility allows for the fabrication of portable and wearable sensing devices.

Silver nanoparticles (AgNPs), synthesized via chemical reduction methods, are widely used as SERS-active materials due to their excellent plasmonic properties and ability to amplify Raman signals [23,24]. The combination of these advanced polymeric substrates with AgNPs enables the development of highly sensitive, stable, and versatile SERS sensors suitable for a wide range of analytical and biomedical applications.

The key elements of the SERS sensors developed in this study are silver nanoparticles (AgNPs), which were synthesized by reducing silver nitrate (AgNO_3_) using sodium borohydride (NaBH_4_) on the surface of a non-woven PAN material. No stabilizers were implemented in order to eliminate the effects of these substances on the spectra of the analyzed materials. The proposed method is quick and inexpensive, eliminating the need for time-consuming sample preparation. As a result, an active substrate of 1 × 1 cm was obtained, which could be easily divided into smaller units for measurements. The enhancement factor of the resulting SERS sensor was evaluated using 4-MBA. Its ability to detect glucose concentrations between 1 and 10 mM was verified using a GOx-modified SERS system.

## 2. Materials and Methods

### 2.1. Materials

Silver nitrate (AgNO_3_, ≥99%), 4-mercaptobenzoic acid (4-MBA, technical grade 90%), Rhodamine B (≥95% purity, 479.01 Da), ethanol (≥95%), D-(+)-glucose (C_6_H_12_O_6_, 180.16 Da, ≥99.5%), sodium borohydride (NaBH_4_), and polyacrylonitrile (PAN, M_w_ = 150 kDa) were purchased from Sigma-Aldrich (St. Louis, MO, USA). Dimethylformamide (DMF) was provided by Reachem (Moscow, Russia). Glucose oxidase was obtained from Biopreparat (Moscow, Russia). Deionized (DI) Milli-Q water (18.2 MΩ·cm) was used in the experiments.

### 2.2. Synthesis and Stability Assessment of Silver Nanoparticles

Silver nanoparticles (AgNPs) were synthesized via the chemical reduction of silver nitrate (AgNO_3_) using sodium borohydride (NaBH_4_) as a reducing agent at 0 °C. This method was subsequently employed for the surface modification of a non-woven polyacrylonitrile (PAN)-based material (see Section 2.4). The aim of this step was to evaluate the temporal stability of the nanoparticles, their behavior upon dilution, and their response to changes in pH, in order to identify optimal conditions for PAN surface coating.

The synthesis was carried out by slowly adding 200 μL of an ice-cold NaBH_4_ solution (2 mM) to 200 μL of an ice-cold AgNO_3_ solution (1.5 mM) under constant stirring. All solutions were prepared using deionized Milli-Q water (18.2 MΩ·cm). Immediately after synthesis, the colloidal solution was transferred to a UV-STAR 96-well microplate (Greiner, Kremsmünster, Austria), and the absorption spectra were recorded using a CLARIOstar Plus microplate reader (BMG Labtech, Ortenberg, Germany).

To assess the effect of dilution, deionized water was added to the freshly prepared AgNP solution in volume ratios of 1:1, 1:2, and 2:1.

To study the effects of the ionic strength and pH, 100 μL NaOH solutions at various concentrations (1 M, 0.1 M, 0.01 M, and 0.001 M) were added to 400 μL of the AgNP dispersion.

### 2.3. Obtaining the PAN Material by Electrospinning

The solution was prepared by dissolving PAN in DMF at a concentration of 130 mg/mL. The mixture was stirred on a magnetic stirrer for three hours, with the first 30 min at 50 °C and the remaining time at room temperature. Every hour, the stirring speed was increased by 150 rpm, starting at 350 rpm. After the mixture had been thoroughly stirred, it was transferred to a syringe attached to a syringe pump. The tip of the syringe was positioned 20 cm away from a fixed substrate, which was a piece of white paper. The rate of solution flow from the syringe was set to 1 mL per hour. The process lasted for 15 min, during which the voltage of the high-voltage power supply was set to 53 kV.

### 2.4. Providing Sensory Capabilities to the Non-Woven Material

#### 2.4.1. Synthesis of SERS Substrates

**Figure 1:**
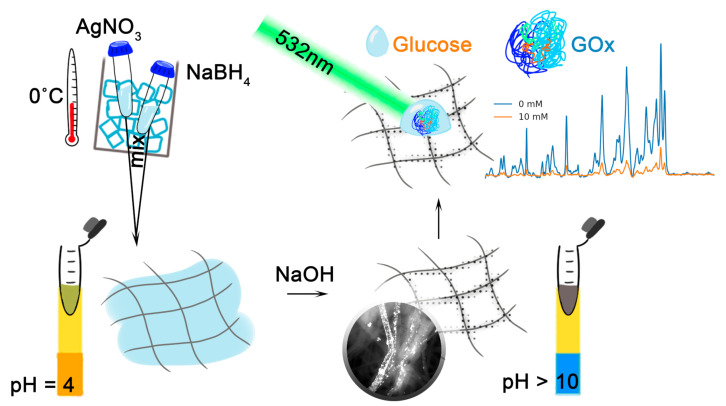
Schematic of AgNP reduction using the borohydride method on a PAN non-woven material, followed by analyte deposition.

To impart sensory properties to the non-woven material made of PAN, a borohydride method was used to obtain AgNPs and their aggregates on the fiber surfaces (Figure 1). In this process, the NaBH_4_ and AgNO_3_ solutions were pre-cooled to 0 °C. Such cooling prevents the formation of overly large particles or unwanted aggregation outside the fibers. PAN is placed in a container with dimensions of 1 × 1 × 1 cm, and 200 μL of the cooled NaBH_4_ solution is added over 5 min (2 mM for samples BH1–BH3 and 20 mM for BH4–BH6). Then, 200 μL of the cooled AgNO_3_ solution is introduced (1.5 mM for BH1–BH3 and 15 mM for BH4–BH6). The volume of the added liquid was determined experimentally to ensure that the entire non-woven material was covered and that reduction occurred on the surface. After a specific exposure time (1 min for BH1 and BH4, 10 min for BH2 and BH5, 30 min for BH3 and BH6), a 1 M NaOH solution was added for 5 min, which changed the pH of the medium from 4 to 10 and adjusted the ionic strength.

Thus, the following SERS substrates were obtained:

- BH1, PAN + NaBH_4_ (2 mM) + AgNO_3_ (1.5 mM) + NaOH (after 1 min, 1 M);
- BH2, PAN + NaBH_4_ (2 mM) + AgNO_3_ (1.5 mM) + NaOH (after 10 min, 1 M);
- BH3, PAN + NaBH_4_ (2 mM) + AgNO_3_ (1.5 mM) + NaOH (after 30 min, 1 M);
- BH4, PAN + NaBH_4_ (20 mM) + AgNO_3_ (15 mM) + NaOH (after 1 min, 1 M);
- BH5, PAN + NaBH_4_ (20 mM) + AgNO_3_ (15 mM) + NaOH (after 10 min, 1 M);
- BH6, PAN + NaBH_4_ (20 mM) + AgNO_3_ (15 mM) + NaOH (after 30 min, 1 M).

#### 2.4.2. Selection of the Alkali Concentration

Before choosing 1 M NaOH, in situ experiments were carried out using the same concentrations as those used in Section 2.2.

The increased ionic strength led to the compression of the electrical double layer around the silver nanoparticles, reducing their colloidal stability and promoting their aggregation.

### 2.5. Characterization of PAN-Based Plasmonic Substrates

The morphologies of the prepared plasmonic substrates were analyzed using scanning electron microscopy (SEM). The SEM images were recorded on a MIRA II LMU instrument (Tescan, Czech Republic) in secondary and backscattered electron modes.

### 2.6. 4-MBA Detection

The model substance 4-MBA was used to evaluate the enhancement factor (EF) of the obtained SERS platforms (Equation (1)). Solutions of 4-MBA in ethanol were prepared at concentrations of 10^−2^ M (Raman) and 10^−4^ M (SERS). Then, 1 μL solution was added to the substrate.

(1) $$EF=\frac{I_{SERS}\cdot c_{R}\cdot p_{R}\cdot t_{R}}{I_{R}\cdot c_{SERS}\cdot p_{SERS}\cdot t_{SERS}},$$

where I—intensity of Raman/SERS band, c—concentration of analyte, p—laser power, t—acquisition time, R—Raman.

### 2.7. Functionalization of GOx Sensors

To impart specificity for glucose detection, the obtained substrates were coated with GOx. The substrates (1 × 1 cm) were divided into 4 parts, and 10 μL of an aqueous GOx solution (1 mg/mL) was applied to each part. The enzyme solution completely covered the substrate sample. Then, the modified substrates were dried at 37 °C.

Then, aqueous glucose solutions with concentrations of 1, 2, 5, and 10 mM were prepared. For each measurement, 10 μL of an aqueous glucose solution was deposited onto the SERS sensors functionalized with GOx.

### 2.8. Raman and SERS Measurements

Raman and surface-enhanced Raman spectroscopy measurements were performed using a Renishaw inVia spectrometer (Renishaw, UK). For the substrates obtained by the borohydride method, a laser with a 532 nm excitation wavelength was used, with a 50×/0.5 N.A (Leica N PLAN L) objective. For each substrate type, a 30 × 30-point map was recorded with a step size of 1 μm, a signal accumulation time of 1 s/spectrum, and laser power of 0.1% (25 μW). The same parameters were used in SERS measurements, except for sample BH4-6 + GOx + glucose, for which the laser power was reduced to 0.05% (12.5 μW). The Raman spectra of 4-MBA (10^−2^ M) were collected with bare PAN, a 10 × 10-point map, laser power of 10% (2.5 mW), and 3 s/spectrum.

The spectra were acquired using diffraction grating of 1200 lines/mm. Measurements were conducted for the following samples: PAN, PAN + Ag, PAN + 4-MBA, PAN + Ag + 4-MBA, PAN + Ag + GOx, PAN + Ag + GOx + glucose.

The Raman spectra of Rhodamine B (10^−5^ M) were recorded using a laser with 785 nm excitation with diffraction grating of 1800 lines/mm. Measurements were performed on a 10 × 10-point map with a step size of 1 μm, an acquisition time of 10 s/spectrum, and laser power of 0.1% (25 μW).

### 2.9. Data Processing

The analysis of the obtained data was conducted in Jupyter Notebook v.6.4.5 (Python 3). Data loading was performed using WDFReader from the renishawWiRE package (v.0.1.16).

4-MBA measurements: The data were centered. After processing the mapping results, principal component analysis was applied. The method was implemented using the decomposition. PCA tool from the scikit-learn package [35]. Matplotlib (v.3.4.3) was used to construct the resulting component distribution maps and spectra.

Glucose measurements: Prior to analysis, the polynomial background was removed using polyfit from the NumPy library (v.1.24.3), and normalization was performed using preprocessing.normalize from the scikit-learn library (v.1.6.1). For further analysis, the data were split into training and test sets using model_selection.train_test_split from scikit-learn (with a training-to-test set ratio of 4:1). Then, the hyperparameters for the gradient boosting regression model, such as the maximum depth of each individual decision tree estimator and the number of estimators in the ensemble, were tuned using GridSearchCV from scikit-learn.model_selection. The fitting of GridSerachCV was performed on the training dataset using 3-fold cross-validation. As a performance metric, the determination coefficient (R^2^) was implemented. The obtained optimal hyperparameters were used in both classification and regression gradient boosting models. A more detailed description of the principles of this analysis, applied to a different dataset, can be found in the article [36]. The performance of the trained classification and regression models was evaluated using the accuracy, confusion matrix (classification), and coefficient of determination (regression), calculated on the test dataset, with accuracy_score, confusion_matrix, and r2_score from sklearn.metrics.

The fitted gradient boosting regression model was implemented to calculate the limit of detection (LoD) of glucose (Equation (2)):

(2) $$LoD=\frac{3.3\cdot \sigma_{blank}}{S},$$

where $\sigma_{blank}$—standard deviation of predictions for blank samples (no glucose), *S*—sensitivity (slope of the calibration curve at concentrations above zero), 3.3—confidence factor (for 95% confidence, derived from *t*-statistics).

Rhodamine B measurements: Each spectrum in the map was processed by removing cosmic ray artifacts (median filter), subtracting the baseline (asymmetric least squares via pybaselines), and applying Savitzky–Golay smoothing. The resulting spectra were normalized to the [0, 1] range. Peaks were automatically detected using find_peaks from scipy.signal (scipy v.1.13.1), filtered by height, prominence, and distance, and plotted with corresponding Raman shifts using matplotlib.

## 3. Results and Discussion

### 3.1. Spectral Changes in AgNPs over Time and When Diluted with Water

**Figure 2:**
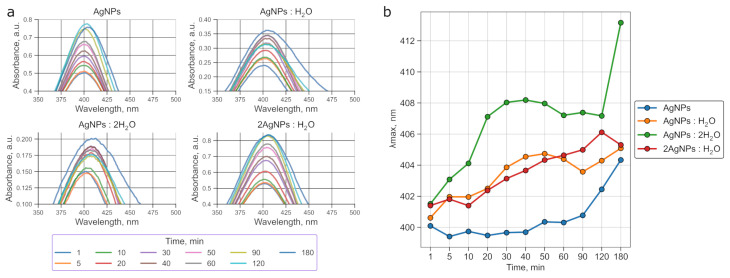
Changes in the absorption spectrum of the silver nanoparticle solution over time, upon dilution with water (1:1, 1:2, 2:1) (**a**). The dependence of the absorption peak position on time (**b**).

The initial silver nanoparticle solution prepared with the borohydride method is quite stable, with the peak shifting by 4 nm and a negligible increase in the full width at half maximum (FWHM, from 60 to 61 nm) over 3 h (Figure 2a). This indicates that the colloidal solution retains its optical properties over time and can be used at various stages after synthesis.

Adding water to the initial solution in different ratios (1:1, 1:2, and 2:1) disrupts nanoparticle growth, causing the peak to broaden and a bathochromic shift (Figure 2b) to occur. For example, in the 1:2 dilution (AgNPs:2H_2_O), the absorption maximum shifts by 11 nm (from 402 to 413 nm), accompanied by a substantial FWHM increase from 51 to 140 nm (Table 1). These changes are attributed to the aggregation-induced growth of nanoparticles and alterations in the local dielectric environment around them.

It is known that repulsive electrostatic forces between AgNPs stabilized by an adsorbed borohydride layer help to maintain their colloidal stability and prevent aggregation in solution [37]. For our purposes, it was crucial to preserve the AgNPs in their monodisperse form until triggering their controlled assembly on fibers via NaOH addition.

To ensure the high stability and uniformity of the nanoparticles, we used only the undiluted colloidal solution, which was stored for no longer than 30 min prior to use. This approach minimized spontaneous aggregation in the bulk solution and allowed us to initiate and localize particles directly on the PAN surface.

### 3.2. The Effects of Alkali on AgNPs

**Figure 3:**
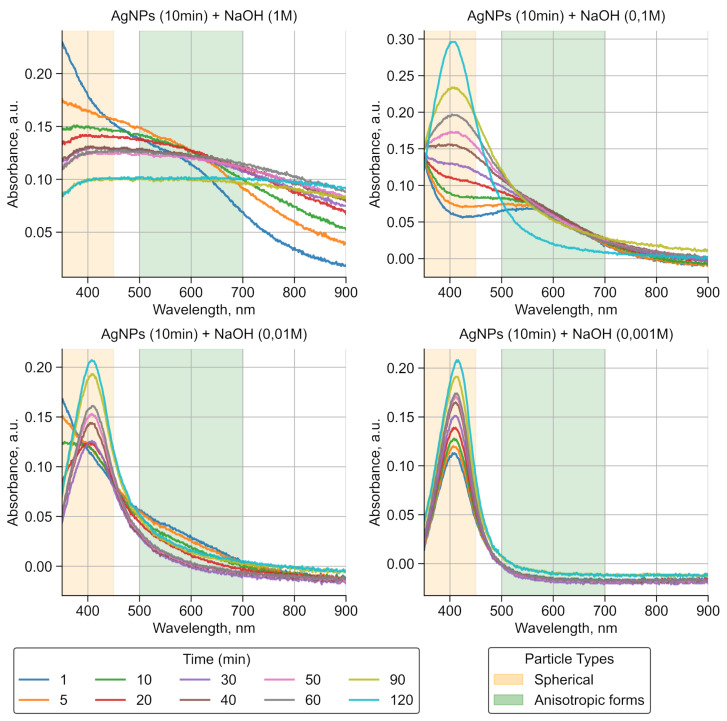
Effects of NaOH concentration on the absorption spectra of silver nanoparticles, with areas where peak appearance is expected marked in yellow (for spherical) and green (for anisotropic).

Upon adding 1 M, 0.1 M, and 0.01 M NaOH solutions, a peak appears in the absorption spectrum in the 500–700 nm range at the initial time point (Figure 3). This peak indicates the presence of longitudinal and transverse plasmonic resonance. After adding 1 M NaOH, the particles do not return to their original form. This is explained by the fact that, at a high pH, spherical nanoparticles deform into more complex shapes and aggregates, affecting their optical properties. Additionally, it is observed that, when less concentrated solutions are used, the aggregation process occurs more slowly, allowing for the formation of nanoparticles with better controlled sizes and shapes. For the sample with 0.1 M NaOH, after 30 min of addition, silver spheres reappear in the solution, but with larger sizes, and the peak in the 500–700 nm region becomes less intense. By 120 min, the plateau disappears completely. For 0.01 M NaOH, this process occurs within 20 min. The lowest concentration of NaOH (0.001 M) does not promote the formation of other particle forms or aggregation.

Given that the remaining colloid cannot be reused once deposited via borohydride reduction on the PAN substrate, we selected 1 M NaOH to induce the rapid and complete aggregation of AgNPs directly on the non-woven fabric surface. This high-pH treatment ensures the immediate collapse of the electrical double layer and the irreversible fusion of the particles, maximizing the substrate coverage in a single step.

### 3.3. Structural Characterization

**Figure 4:**
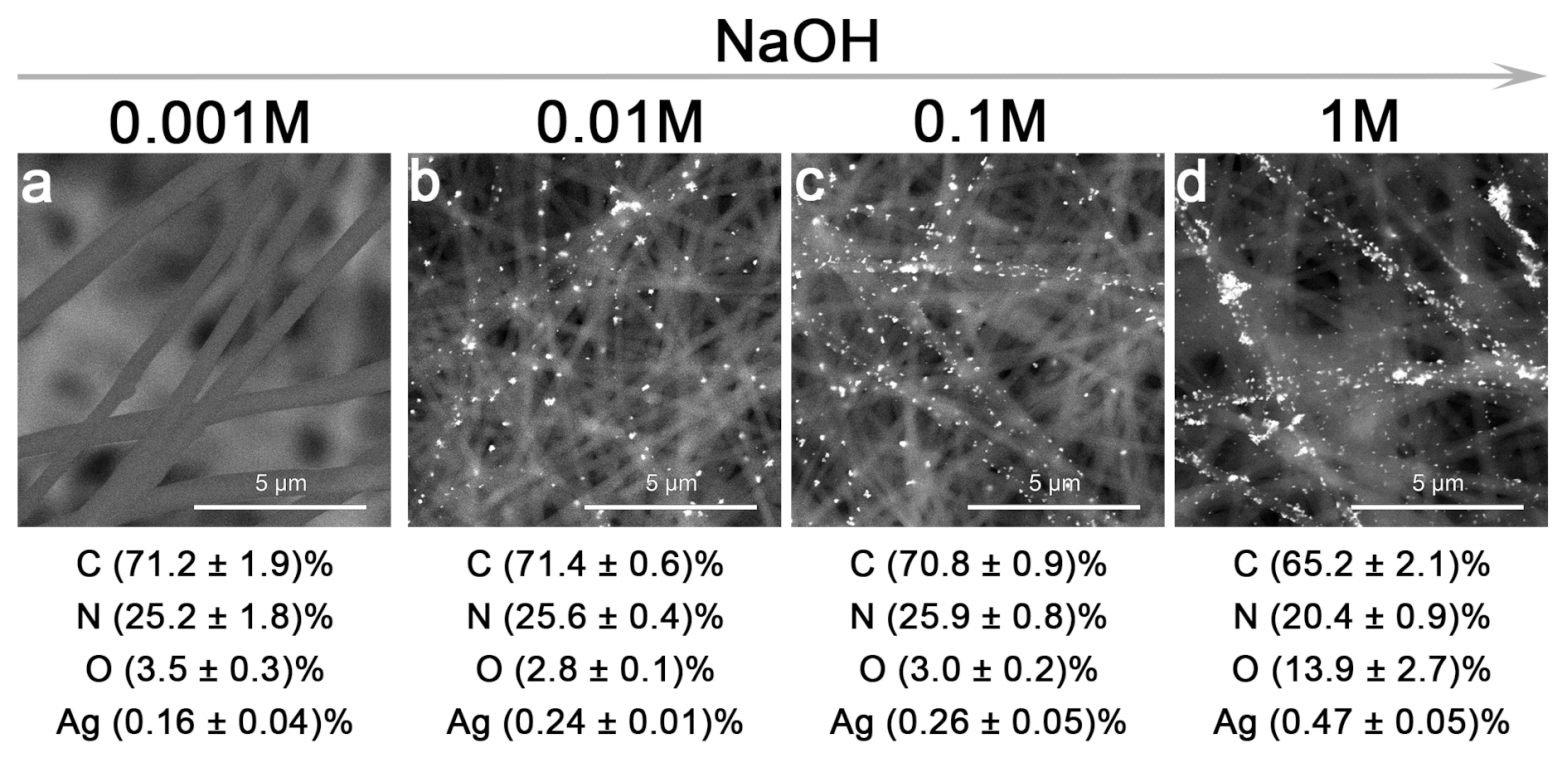
SEM images in the electron backscattering mode for substrates obtained using different concentrations of NaOH: 0.001 M (**a**), 0.01 M (**b**), 0.1 M (**c**), 1 M (**d**). The EDX data are shown below the image. Scale bar is 5 μm.

With the rise in the concentration of alkali, the quantity of silver on the filaments increases. The high percentages of C, N, and O are related to the composition of polyacrylonitrile and the location of the non-woven fabric on the conductive adhesive tape. A 1 M NaOH solution was chosen for further use, as it is the most effective for the formation of “hot spots” (Figure 4).

PAN plays a crucial role in determining both the spatial distribution of AgNPs and the resulting SERS effect. Owing to its fibrous and porous non-woven structure, it provides a high-surface-area scaffold that facilitates the nucleation and immobilization of AgNPs. In addition, the presence of nitrogen-containing functional groups (–C≡N) in PAN can provide coordination sites, promoting the uniform adsorption of silver ions (Ag^+^) prior to chemical reduction.

**Figure 5:**
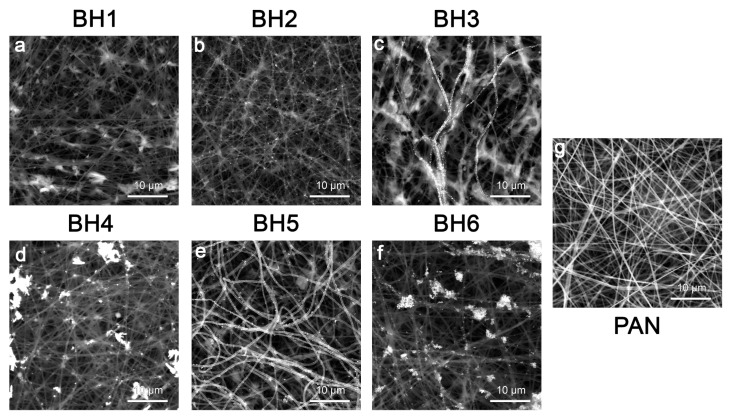
SEM images for SERS substrates BH1-6 (**a**–**f**) and PAN without Ag (**g**). Scale bar is 10 μm. Backscattered electron mode was implemented.

The SEM images (Figure 5) reveal that AgNPs preferentially accumulate along the surface contours of individual PAN fibers. This fiber-guided distribution supports the formation of interparticle junctions and nanogaps—“hot spots”—which are essential in achieving significant electromagnetic field enhancement in SERS.

In this work, six types of SERS substrates were obtained with the reduction of AgNPs by the borohydride method on the surface of a PAN non-woven material (see Figure 1 for schematics; Figure 5). The concentrations of NaBH_4_ and AgNO_3_ used in the reduction process were 10 times greater for BH4-6 samples than for BH1-3 samples. The reduction time increased for both the BH1-BH3 sequence and the BH4-BH6 sequence. Details are provided in Section 2.4. With an increase in the reduction time, there was an increase in AgNP coverage on individual fibers, especially for the BH1-3 sequence. The irregular reduction of silver in BH4-6 samples is related to the concentrations used, presumably due to particle nucleation and growth being disrupted.

### 3.4. Surface-Enhanced Raman Spectra (SERS) from 4-Mercaptobenzoic Acid (4-MBA)

**Figure 6:**
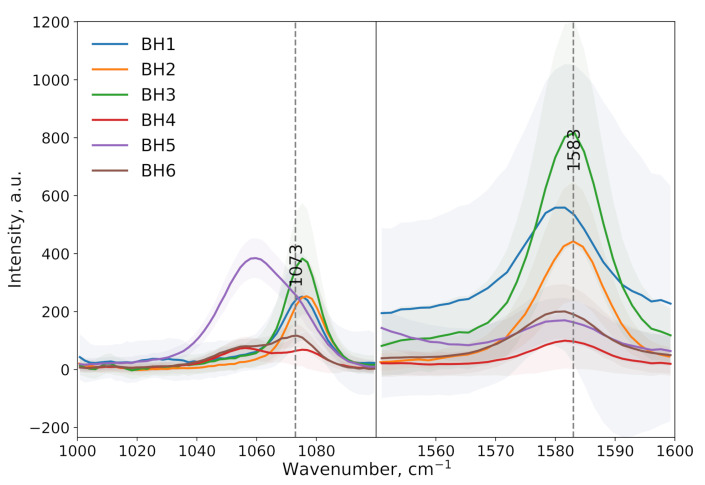
Most significant regions of the SERS spectra of 4-MBA (10^−4^ M) recorded on the surfaces of silver-functionalized PAN substrates, obtained via the borohydride method.

SERS spectra were obtained for a series of samples (BH1–BH6) upon detecting the model compound 4-MBA (Figure 6). For samples BH1–BH3, characteristic peaks of 4-MBA were observed at ∼1073 cm^−1^ (corresponding to ν(CS) + δ(CCH) vibrations) and ∼1583 cm^−1^ (aromatic ring ν(CC) vibrations), indicating stable signal enhancement on these substrates. The enhancement factors (EFs) for these samples ranged from 1.2 × 10^4^ to 3.2 × 10^4^ at 1073 cm^−1^ and from 4.9 × 10^4^ to 1.8 × 10^5^ at 1583 cm^−1^, demonstrating the high sensitivity of the substrates to 4-MBA detection (Table 2).

Previously, we synthesized SERS substrates through the chemical reduction of silver nitrate [34]. These substrates exhibited higher EFs, which was attributed to their more developed morphologies. The novel approach involving sodium borohydride demonstrates a remarkable capacity for the overgrowth of minute silver structures directly onto the filaments, coupled with the capability to meticulously regulate their packing density.

However, for samples BH4–BH6, anomalies were observed in the spectra:

- The shifting and broadening of characteristic peaks toward the shorter-wavelength region (hypsochromic shift), which may be related to changes in the chemisorption of the thiol group (−SH) on the nanoparticle surface or conformational changes in the molecule;
- The appearance of an additional peak in BH4 and BH5, likely due to the interaction of 4-MBA with impurities or the inhomogeneity of the substrate coating.

Due to these deviations, samples BH4–BH6 were excluded from further analysis in glucose detection, as the altered spectral characteristics could have hindered the accurate identification and quantitative evaluation of the signal. For BH1–BH3, the spectra matched the expected response for 4-MBA, confirming the suitability of these substrates for subsequent biomolecule detection experiments.

To further evaluate the analytical performance of the optimized substrates, we examined their SERS responses to Rhodamine B (RhB, 10^−5^ M), a model dye molecule widely used for SERS substrate benchmarking, alongside 4-MBA. As shown in Figure S1 (SI), BH2 substrates demonstrated characteristic RhB vibrational bands at 620 cm^−1^ (C−C−C bending), 1356 cm^−1^ (N−C−H bending), and 1643 cm^−1^ (C=O stretching), confirming their capability for the sensitive detection of dye molecules.

### 3.5. Glucose Detection

When glucose is present, GOx immobilized on silver nanoparticles catalyzes its oxidation to gluconic acid, with H_2_O_2_ produced as a byproduct. The accumulation of gluconic acid near the sensor surface causes a localized pH decrease, shifting the net charge of the enzyme toward its isoelectric point.

This shift reduces the electrostatic interactions between GOx and the silver scaffold, resulting in the partial desorption of the enzyme into the surrounding solution. Consequently, the local surface density of flavin adenine dinucleotide co-factors within the SERS “hot spots” decreases, leading to the attenuation of the characteristic Raman signal. The extent of signal reduction correlates with the glucose concentration, yielding a quantitative “turn-off” response [38,39]. This mechanism is consistent with previous reports on GOx-functionalized silver-based glucose sensors [27].

In order to analyze the SERS spectra obtained from glucose sensing, regression and classification models based on gradient boosting were implemented. Gradient boosting is an ensemble model that uses the sequential fitting of individual decision trees. The main hyperparameters for this type of model are the maximum depth of each tree and the number of trees in the ensemble. To optimize the models, a grid search of these hyperparameters was performed using three-fold cross-validation. The maximum depth and the number of estimators were varied between 1 and 5 and 50 and 350, respectively. The best hyperparameters, as determined by the R^2^ metric, were max_depth = 3 and n_estimators = 200. These values were used in both the classification and regression models.

**Figure 7:**
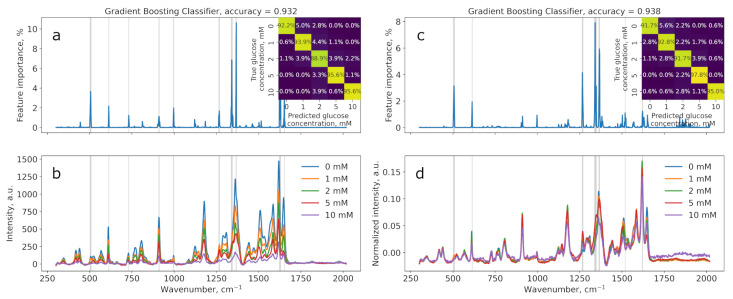
Results of the classification model using gradient boosting on BH2: non-normalized (**a**,**b**) and normalized (**c**,**d**). Feature importances plots with significant wavenumbers (importance > 1%) indicated with grey lines (**a**,**c**). Classification confusion matrices in insets (**a**,**c**).

**Figure 8:**
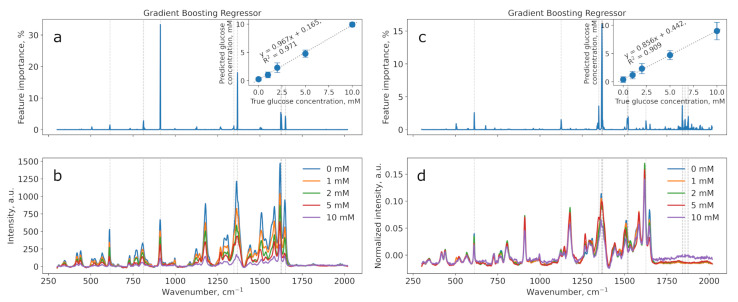
Results of the regression model using gradient boosting on BH2: non-normalized (**a**,**b**) and normalized (**c**,**d**). Feature importances plots with significant wavenumbers (importance > 1%) indicated with grey lines (**a**,**c**). Calibration lines for regression models in insets (**a**,**c**).

The results of applying regression and classification models based on gradient boosting to data obtained from measurements on substrates produced by the borohydride method (BH2) are presented in Figure 7 and Figure 8.

Both datasets show that the model successfully predicts the glucose concentration, as evidenced by the high accuracy coefficients and corresponding R^2^ values.

Table 3 contains the performance scores for two gradient boosting models: regression (R^2^) and classification (accuracy). These values are provided for both normalized and non-normalized data and were measured for three substrates (BH1, BH2, BH3). The graphs for samples BH1 and BH3 are shown in Figures S2–S5, SI.

In most cases, normalizing the data improves the model’s performance. This improvement may be attributed to the fact that normalization helps to eliminate differences in feature scales, making the model training more stable and efficient. An exception is substrate BH2 when using GradientBoostingRegressor, where normalization decreases the model’s performance. This could be due to non-normalized data better reflecting the true dependencies in the original data for this particular substrate.

GradientBoostingClassifier demonstrated higher classification accuracy compared to the R^2^ scores of GradientBoostingRegressor across almost all substrates except BH2, suggesting that the discrete classification task (predicting concentration classes) was better captured by the classifier under these experimental conditions. However, the regression model is preferable for glucose sensing, as there is a need not only to decide whether the glucose concentration is in the normal range or higher but also to determine the exact value in order to take appropriate actions.

As the best-performing SERS substrate was found to be BH2, the limit of detection for glucose was also evaluated based on Equation (2) and found to be 0.66 mM.

The BH2 sample was also covered with artificial sweat to simulate the use of the substrate under real-life conditions (SI, Figure S6). It was observed that the salts present in the composition affected the distribution of “hot spots”. For further use, the system needs to be optimized, with denser silver growth on the fibers to prevent the sweat salt crystals from disrupting the material’s structure.

In order to highlight the benefits of the proposed AgNP-based SERS substrates, a comparison should be performed (Table 4).

Compared to other AgNP-based SERS substrates, our PAN non-woven platform synthesized via in situ borohydride reduction demonstrates several notable features.

- Simplicity and Scalability: Most referred methods for SERS substrate fabrication are either more time-consuming and complex [40,41] or less homogenous at a micro level [42]. The in situ reduction method is rapid, does not require stabilizers, and is easily scalable for large-area substrates.
- Enhancement Factor and Sensitivity: While the EF (1.8 × 10^5^) is comparable to or slightly lower than those of some other platforms, our substrate achieves a competitive LoD for glucose (0.66 mM) within the physiologically relevant range, making it suitable for non-invasive sensing. Although the EF and LoD were slightly lower than in our previous publication [34], the R^2^ coefficient for the gradient boosting regression model is much higher (0.971 compared to 0.756 in [34]).
- Application: The PAN substrate’s chemical and mechanical stability, combined with enzyme functionalization, enables indirect glucose detection with high selectivity.
- Cost and Practicality: The materials (PAN, AgNO_3_, NaBH_4_) are low-cost and widely available, making the approach attractive for disposable or point-of-care SERS devices.

Further optimization of the AgNP loading and substrate morphology may bridge the gap in the EF while maintaining the simplicity and scalability of the current approach.

## 4. Conclusions

In this study, we report the borohydride-mediated in situ synthesis of silver nanoparticles on electrospun PAN fibers, which yields highly reproducible and tunable SERS substrates. By systematically varying the concentrations of NaBH_4_ (2–20 mM) and AgNO_3_ (1.5–15 mM), adjusting the reduction times between 1 and 30 min, and applying a 1 M NaOH post-treatment, we achieved a controllable nanoparticle morphology. UV–Vis spectroscopy revealed a plasmon resonance peak at ∼400 nm that shifted by 4 nm over three hours, indicating excellent colloidal stability.

Morphological analysis by SEM confirmed that higher reagent concentrations and longer reduction times produced denser, more irregular AgNP coatings, which translated directly into enhanced SERS performance, but the signal became less reproducible or uncharacteristic peaks appeared. Using 4-MBA (10^−4^ M) as a probe molecule, the EF for the BH1–BH3 substrates ranged from 1.2 × 10^4^ to 3.2 × 10^4^ at 1073 cm^−1^ and from 4.9 × 10^4^ to 1.8 × 10^5^ at 1583 cm^−1^. Among these, the BH2 substrate—prepared with 2 mM NaBH_4_, 1.5 mM AgNO_3_, and 10 min reduction—exhibited optimal performance with EFs of 2.4 × 10^4^ (1073 cm^−1^) and 1.1 × 10^5^ (1583 cm^−1^).

Building on this platform, we functionalized the BH2 substrates with GOx to create biosensors for glucose detection. SERS spectra recorded in the 0–10 mM glucose range showed clear calibration curves exhibiting excellent linearity (R^2^ = 0.971). Data normalization generally improved model performance, except for one substrate (BH2), where the raw data better preserved the concentration-dependent trends. The calculated limit of detection for glucose was found to be 0.66 mM.

The optimized sensor design, particularly using nanostructured substrates, shows promise for non-invasive glucose monitoring. Further refinement of substrate fabrication could enhance its sensitivity and robustness for real-world applications, such as wearable diabetes management devices.

## Figures and Tables

**Table 1:** FWHM of the AgNP absorption peak at 1 and 180 min for different water dilution ratios.

Sample	FWHM at 1 min, nm	FWHM at 180 min, nm
AgNPs	60	61
AgNPs–H_2_O	61	71
AgNPs–2 H_2_O	51	140
2 AgNPs–H_2_O	69	71

**Table 2:** Enhancement factors of SERS spectra of 4-MBA (10^−4^ M) for characteristic peaks.

Sample	1073 cm^−1^	1583 cm^−1^
BH1	2.4 × 10^4^	1.4 × 10^5^
BH2	2.4 × 10^4^	1.1 × 10^5^
BH3	3.2 × 10^4^	1.8 × 10^5^
BH4	1.2 × 10^4^	4.9 × 10^4^
BH5	2.9 × 10^4^	1.1 × 10^5^
BH6	2.3 × 10^4^	1.3 × 10^5^

**Table 3:** Metrics of gradient boosting models in glucose measurements on substrates BH1–3.

Model (Metric)	Substrate	Normalized Data	Non-Normalized Data
	BH1	0.616	0.543
Regression (R^2^)	BH2	0.909	0.971
	BH3	0.831	0.737
Classification	BH1	0.697	0.558
(accuracy)	BH2	0.938	0.932
	BH3	0.706	0.704

**Table 4:** Analysis of the proposed SERS substrate in comparison with other SERS substrates based on silver nanoparticles.

Substrate	AgNP Synthesis	EF	LoD	Application	Reference
PAN non-woven	In situ borohydride reduction	1.8 × 10^5^ (4-MBA)	0.66 mM (glucose)	4-MBA, RhB, glucose (indirect)	Current article
PAN non-woven	In situ silver mirror reaction	2.5 × 10^6^ (4-MBA)	0.5 mM (glucose)	4-MBA, glucose (indirect)	[34]
Agarose hydrogel	Physically induced colloidal AgNP aggregates	1.4 × 10^7^ (malachite green)	5 nM (malachite green)	Nile blue, crystal violet, malachite green	[40]
Glass surface	Deposition of chemically reduced Ag nanostars in layers	2.9 × 10^4^ (imidacloprid)	3.9 mM (imidacloprid)	Pesticide detection	[41]
Filter paper	Dipping into AgNP suspension	Not mentioned	34 μM (amitriptyline)	Antidepressant sensing	[42]

## Data Availability

The datasets presented in this article are not readily available because of time limitations. Requests to access the datasets should be directed to Ekaterina S. Prikhozhdenko.

## Supplementary Materials

The following supporting information can be downloaded at https://www.mdpi.com/article/10.3390/s25134143/s1. Figure S1: Averaged and normalized SERS spectra of Rhodamine B on BH2 substrate; Figure S2: Results of the classification model using gradient boosting on BH1: non-normalized (left) and normalized (right); Figure S3: Results of the regression model using gradient boosting on BH1: non-normalized (left) and normalized (right); Figure S4: Results of the classification model using gradient boosting on BH3: non-normalized (left) and normalized (right); Figure S5: Results of the regression model using gradient boosting on BH3: non-normalized (left) and normalized (right); Figure S6: SEM image for BH2 covered with sweat. Scale bar is 1 μm. Backscattered electron mode was implemented.

## Abbreviations

The following abbreviations are used in this manuscript:

SERS	surface-enhanced Raman scattering
GOx	glucose oxidase
NP	nanoparticle
PAN	polyacrylonitrile
EF	enhancement factor
4-MBA	4-mercaptobenzoic acid
LoD	limit of detection
SEM	scanning electron microscopy
FWHM	full width at half maximum
