# Supplementary material for: Borohydride Synthesis of Silver Nanoparticles for SERS Platforms: Indirect Glucose Detection and Analysis Using Gradient Boosting
Source: Sensors (Basel). 2025 Jul 3;25(13):4143. doi: 10.3390/s25134143 (PMC12252442; doi:10.3390/s25134143)
Supplement: Supplementary file 1 [file sensors-25-04143-s001.zip › sensors-3667423-supplementary.pdf]

Borohydride Synthesis of Silver Nanoparticles for SERS Platforms: Indirect Glucose Detection, Analysis Using Gradient Boosting

Viktoriia Bakal, Olga Gusliakova, Anastasia Kartashova, Mariia Saveleva, Polina Demina, Ilya Kozhevnikov, Evgenii Ryabov, Daniil Bratashov*, Ekaterina Prikhozhdenko

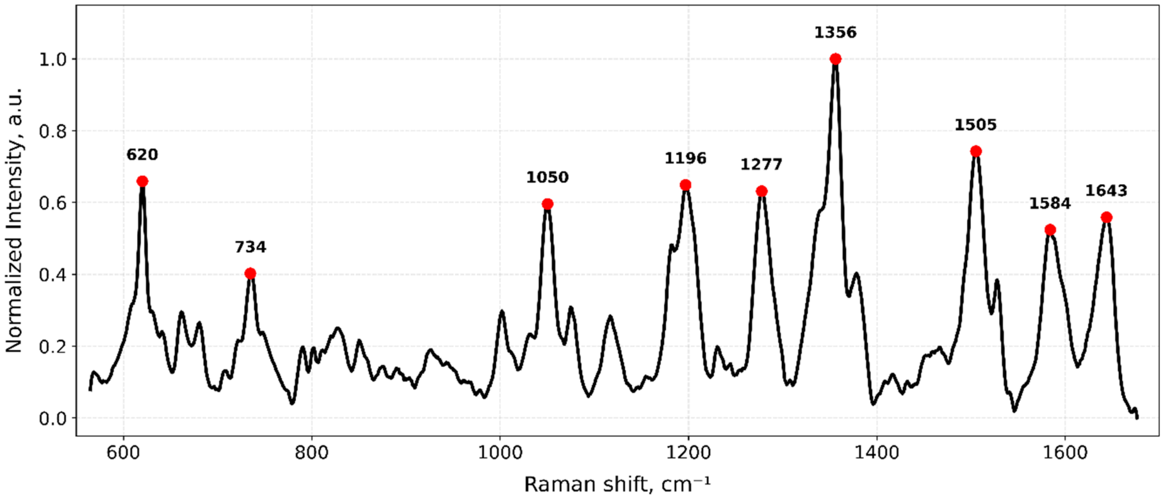

Figure S1. Averaged and normalized SERS spectrum of Rhodamine B on BH2 substrate.

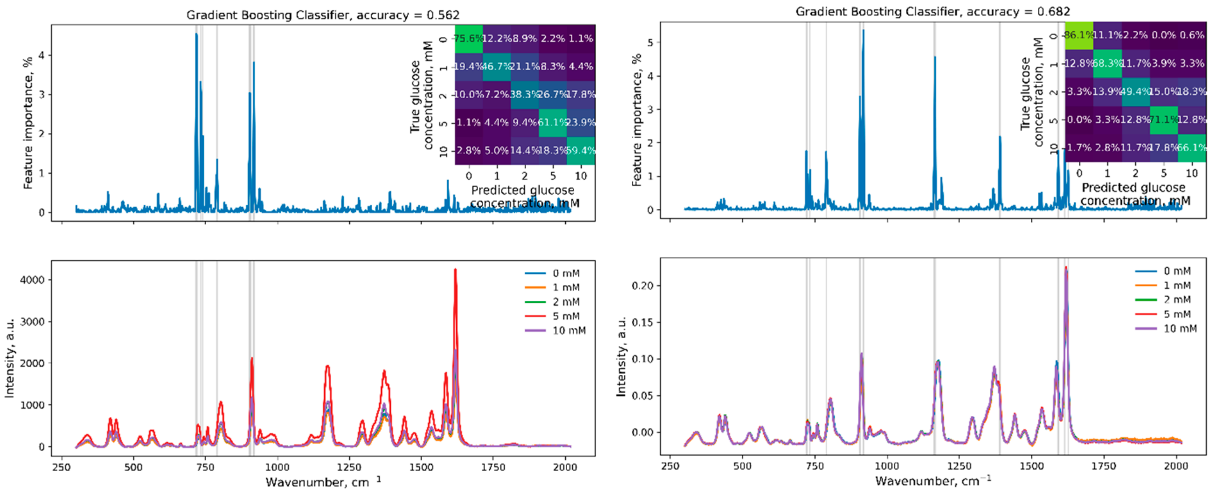

Figure S2. Results of the classification model using the gradient boosting on the BH1: non-normalized (left) and normalized (right).

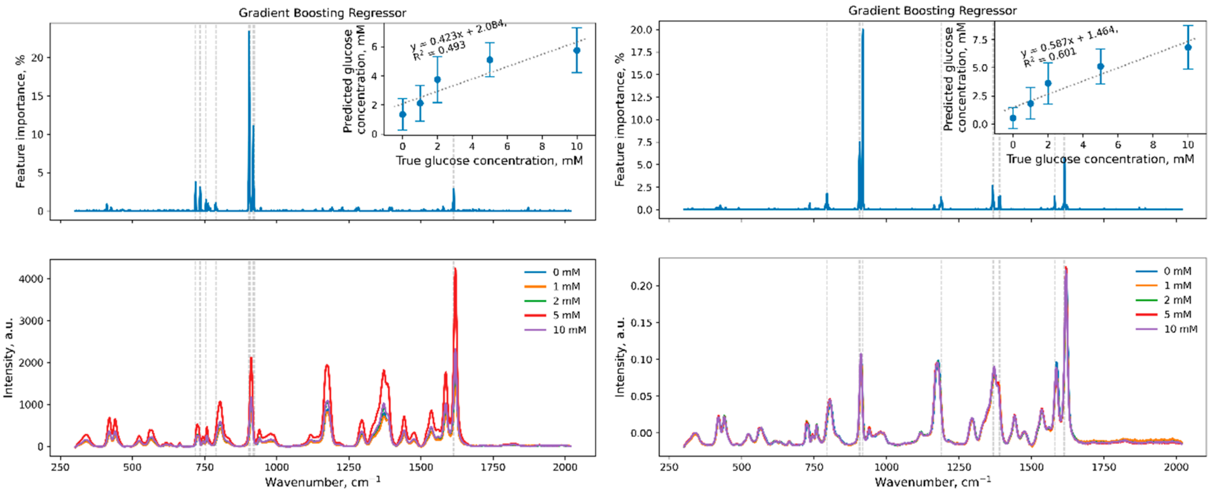

Figure S3. Results of the regression model using the gradient boosting on the BH1: non-normalized (left) and normalized (right).

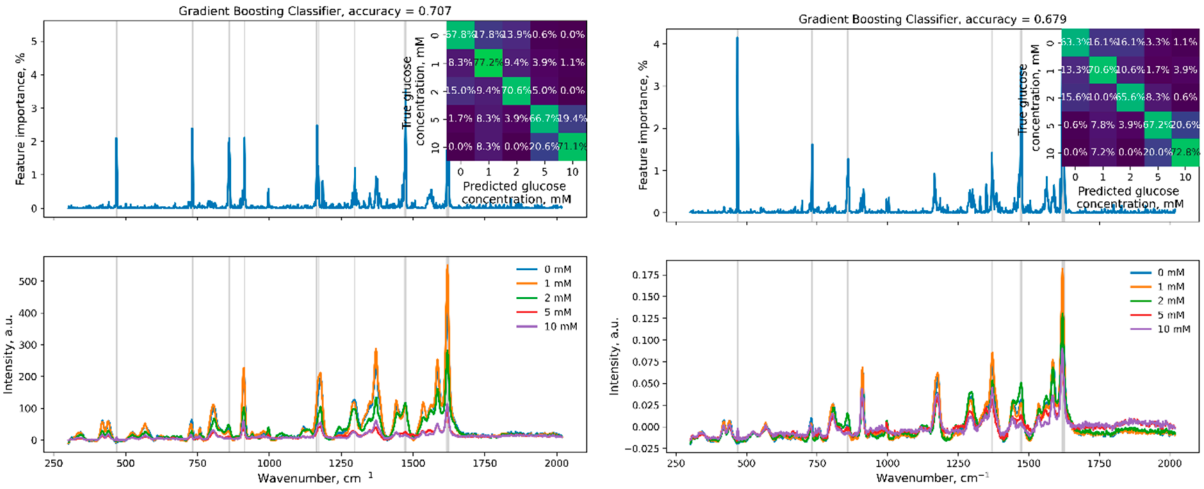

Figure S4. Results of the classification model using the gradient boosting on the BH3: non-normalized (left) and normalized (right).

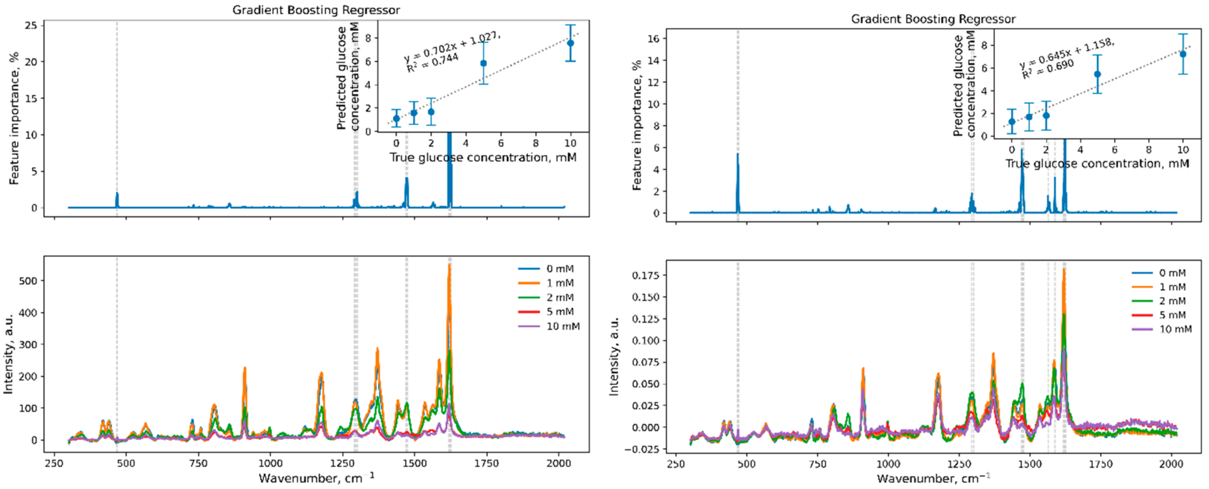

Figure S5. Results of the regression model using the gradient boosting on the BH3: non-normalized (left) and normalized (right).

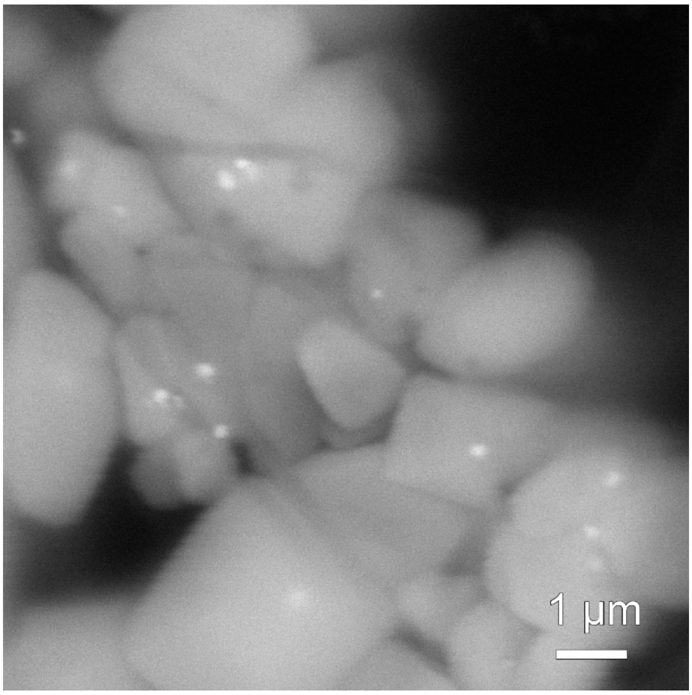

Figure S6. SEM image for BH2 covered with sweat. Scale bar is 1 µm. Back-scattered electron mode was implemented.
